# What Should the Deaf Do?

**Published:** 1889-08-17

**Authors:** Morris Jones

**Affiliations:** Marine-terrace, Aberystwith


### Editor's Letter-Box.

[Our Correspondents are reminded that prolixity is a great bar to publication, and that brevity of style and conciseness of statement greatly facilitate early insertion.]

### WHAT SHOULD THE DEAF DO?

There are thousands who highly value the useful information given in The Hospital on different departments of medicine and surgery—epilepsy, eye, etc. Some account of what is done or can be done in the case of deafness and dumbness as a consequent would be *very* acceptable. There are large numbers in the country suffering in this way. There are large numbers of children deaf or partly, and the parents know not what to do and what can be done. The ordinary general practitioner is, I fancy, very backward in this department. Anything said to enable parents to distinguish between hopeless cases and those that could possibly benefit by going to London, or to some town where there are specialists, would be *very* valuable. There seems to be an increase, at least in some parts of the country, in the number of deaf children, and I am sure any information you may be pleased to give will draw attention to your excellent paper and the hospitals generally. Pardon me for writing thus.—Yours, etc., Morris Jones.

Marine-terrace, Aberystwith, August 8, 1889.

[Some suggestions on this topic will be given next week.—Ed.]

